# Views and experiences of behaviour change techniques to encourage walking to work: a qualitative study

**DOI:** 10.1186/1471-2458-14-868

**Published:** 2014-08-23

**Authors:** Sunita Procter, Nanette Mutrie, Adrian Davis, Suzanne Audrey

**Affiliations:** School of Social and Community Medicine, University of Bristol, Canynge Hall, Whatley Road, Bristol, BS8 2PS UK; Institute for Sport, Physical Education & Health Sciences, The Moray House School of Education, The University of Edinburgh, St Leonard’s Land, Holyrood Road, Edinburgh, EH8 8AQ Scotland; Public Health Support to City Transport, Bristol City Council, City Hall, College Green, Bristol, BS1 5RT UK

**Keywords:** Walking, Behaviour change techniques, Qualitative research, Active travel, Physical activity, Workplace

## Abstract

**Background:**

High levels of physical inactivity are linked to several chronic diseases including coronary heart disease, type-2 diabetes, obesity, some cancers and poor mental health. Encouraging people to be more active has proven difficult. One way to incorporate physical activity into the daily routine is through the journey to and from work. Although behaviour change techniques (BCTs) are considered valuable in promoting behaviour change, there is very little in the published literature about the views and experiences of those encouraged to use them.

**Methods:**

The Walk to Work study was a feasibility study incorporating an exploratory cluster randomised controlled trial. The 10-week intervention involved training workplace-based Walk to Work promoters (volunteers or nominated by participating employers) to encourage colleagues to increase walking during their daily commute. The intervention used nine specific BCTs: Intention formation, barrier identification, specific goal setting, instruction, general encouragement, self-monitoring of behaviour social support, review of behavioural goals and relapse prevention. Digitally recorded interviews were undertaken with 22 employees, eight of whom were Walk to Work promoters to understand their views and experiences of using these techniques. The Framework method of data management and constant comparison were used to analyse the data and identify key themes.

**Results:**

For each individual BCT, there appeared to be people who found it useful in helping them to increase walking to work and others who did not. Following training, the Walk to Work promoters varied in the extent to which they were able to fulfil their role: additional support and encouragement during the 10-week intervention may be required for the promoters to maintain motivation. Wider contextual (economic climate, unprecedented wet weather) and organisational (workload, car parking facilities) issues were identified that influenced the delivery of, and response to, the intervention.

**Conclusions:**

Walk to work interventions employing BCTs should include sufficient techniques to enable participants to choose a ‘package’ to suit their needs. Additional support at organisational level should also be encouraged, and consideration given to wider contextual factors that impinge on the delivery of, and response to, the intervention.

**Trial registration:**

ISRCTN72882329.

**Electronic supplementary material:**

The online version of this article (doi:10.1186/1471-2458-14-868) contains supplementary material, which is available to authorized users.

## Background

Physical inactivity is a risk factor for global mortality with major implications for the prevalence of non-communicable diseases such as coronary heart disease, type-2 diabetes, some cancers and depression [1, 2]. Research has shown that 150 minutes or more of moderate intensity physical activity per week reduces the risk and this has been incorporated into public health physical activity guidelines [3]. Walking is a familiar, convenient and free form of physical activity that most people can incorporate into everyday life and evidence suggests that successful interventions can increase walking among targeted participants by up to 30–60 minutes a week on average [4].

In the UK around 30 million people are classified as being in work [5], which comprises nearly half the population. The most common mode of commuting to work is by car or van (66%), with public transport (17%) and walking (11%) also playing a role [6]. Employing organisations are increasingly encouraged to reduce private car use amongst their employees and increase active commuting (walking and cycling) through Travel Plans, not only for health reasons but also the potential benefits for wider society resulting from a reduction in traffic congestion and improvements to air quality [7]. There are also potential financial benefits for the individual because of reduced commuting costs, and for organisations through a reduction in the need for car parking facilities.

In the UK, public health guidance recommends that employers encourage employees to walk, cycle or use another mode of transport involving physical activity [8]. This guidance also recommends further research on the effectiveness of interventions in different size and types of workplaces and the characteristics of employees (for example age, ethnicity, gender, socioeconomic status or disability).

Schemes to encourage active travel can be considered within a socio-ecological model examining influences at the policy, community, organisational, interpersonal and intrapersonal levels [9] (see Table 1). Behaviour change techniques (BCTs) are the active ingredients within an intervention designed to change behaviour and are observable, replicable and irreducible components that can be used alone or in combination [10]. BCTs to encourage active travel can be considered to be enacted at the intrapersonal and interpersonal levels, whilst being encouraged by employing organisations, supported by social norms and values in the community, and promoted through public policy. In a longitudinal study exploring patterns and predictors of changes in active commuting in Cambridge, UK it was found that the provision of more supportive physical environments for walking, improving public transport and limiting availability of workplace car parking may promote uptake and maintenance of active commuting [11].

**Table 1:** **The socio-ecological model and the Walk to Work intervention**

Socio-ecological level		Walk to Work objective
**Intrapersonal**	Individual knowledge, skills, attitudes, behaviour	Increase employees’ knowledge of the benefits of walking to work
		Identify and address perceived personal barriers
		Personal goal setting
		Change in travel to work routines
		Increase employers’ knowledge of the benefits of walk to work schemes
		Increase employers’ support for employee walk to work schemes
**Interpersonal**	Influence family, friends, work colleagues	Identify and address specific barriers e.g. school run
		Colleagues and friends encourage each other to walk to work
		Increase ‘culture’ of walking to work
**Institutional**	Workplace policies, procedures and facilities	Enhance employer/workplace support for walking to work
**Community**	Built, natural and social environment and local resources	Identify safe, feasible walking routes
		Identify local groups and organisations to support and enhance walking to work
**Public policies**	National and local initiatives, policies and plans	Increase employee and employer understanding of national and local policy context, walking initiatives and websites

Until recently interventions to change individual behaviour have been difficult to replicate as techniques were often not readily identifiable or were poorly defined. A taxonomy of 26 BCT’s was identified in 2008 [12] with subsequent work undertaken to improve labels and definitions and to reach a wider consensus of agreed distinct BCTs [10, 13]. The 2008 taxonomy has been successfully used to categorise the BCTs used in healthy eating and physical activity interventions [14] with ‘self-monitoring’ combined with at least one other technique the most effective. Bird et al. [15] identified 46 walking and cycling controlled interventions targeted at adults and coded the BCTs using the 26 item taxonomy [12]. They reported a statistically significant effect on walking and cycling outcomes with the mean number of BCTs used being 6.43 (SD = 3.92) and the most commonly used techniques ‘self-monitoring’ and ‘intention formation’ [15]. The UK’s National Institute for Health and Care Excellence (NICE) has recently issued recommendations advising that interventions should use BCTs based on goals and planning, feedback and monitoring, and social support [16].

Encouraging walking to work in a workplace environment, where most of the target population will not be classified as ‘ill’ , may be considered the responsibility of workplaces rather than external health promotion specialists. Workplace Travel Plans often incorporate measures to reduce private car use [17]. In addition, peer delivered health promotion has been used [18] to provide colleagues with information, social support and general encouragement.

The effectiveness of interventions to promote active travel tends to be measured using self-report surveys. The Walk to Work feasibility study used objective measures to show walking to work was associated with both higher levels of overall physical activity and moderate to vigorous physical activity (MVPA), compared with those travelling by car [19]. The study also incorporated a process evaluation to examine the context and implementation of the intervention, and participants’ responses to it. Few studies have looked qualitatively at participants’ views and experiences of the BCTs used in interventions. The aim of this paper is to describe the BCTs used during the Walk to Work intervention, delivered by workplace Walk to Work promoters, and examine participants’ and promoters’ views and experiences of the different techniques.

## Methods

### The walk to work study

The aim of the Walk to Work study was to test the feasibility of an employer-led scheme to encourage walking to work. The study took place between November 2011 and October 2013 in the south west of England, with the intervention being implemented in the summer of 2012.

**Figure 1:**
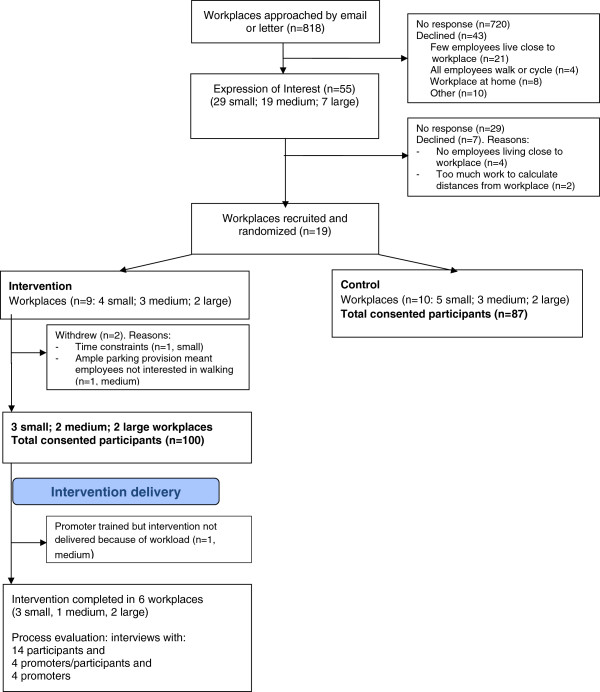
**CONSORT: Walk to Work flow of workplaces and participants.**

An exploratory cluster randomised controlled trial was conducted in 17 workplaces (7 intervention and 10 control). Workplaces were categorised as small (up to 50 employees), medium (1–250 employees) or large (more than 250 employees), and by location (city centre or suburban) and type of business. Workplaces were approached through the local Chambers of Commerce by letter and email for initial expressions of interest including willingness to allocate employee time for study activities. Assignment of workplaces to the intervention group employed computer generated allocation. The flow of workplaces and participants through the study is shown in Figure 1. Data collection included self-report and objective measurement of travel method and physical activity, as well as process evaluation and an assessment of costs. The University of Bristol Faculty of Medicine and Dentistry Committee for Ethics gave ethical approval for the study. We report this qualitative study following the RATS qualitative research review guidelines (http://www.biomedcentral.com/authors/rats).

### The walk to work intervention

Booklets for the promoters and participating employees in the intervention arm were developed by expert members of the research team following a resource review. The review looked at current resources that promote walking (and in particular the benefits of walking to work) which included the public health campaign in England called change4life [20], material published by charities such as Living Streets [21] and Walkit.com [22], material from the ‘Walk in to Work out’ intervention [23] as well as drawing on policy guidance [2, 3, 24].

The booklets included information about the health, environmental, economic and social benefits of walking to work. Each workplace in the intervention arm was asked to identify at least one Walk to Work promoter, either a volunteer or staff member nominated by their employer. Their role was to encourage other participating employees in the organisation to increase walking during their journey to and from work. The promoters were provided with a half-day of training by the researchers on the benefits of walking to work. Nine BCTs from the Abraham and Michie (2008) Taxonomy [12] (Table 2) to be used during the intervention were explained, as was how to implement them. This was supported by a promoter’s booklet outlining how to support participants during four contacts over the 10 weeks of the intervention, and the BCTs to use at each contact point. The precise format for each contact in the workplace (whether individual or in groups, and whether face-to-face, email or telephone) was decided between the promoters and participants. The promoters were encouraged to document the support offered to participants, and any issues raised by employees or employers, in diary pages at the back of the booklets.

**Table 2:** **Behavioural change techniques used during the 10-week Walk to Work intervention**

Contact	BCT [12] NICE categories [16]	Walk to work intervention
1 (week 1) Getting Started	Intention formation (Goals and planning)	Employee decides to participate in the Walk to Work intervention and try to increase the amount of walking during the journey to and from work.
	Barrier identification (Goals and planning)	Promoter works with participant to determine the benefits and barriers of walking to work and some proposed solutions. Participant’s booklet contains some examples of barriers and possible solutions.
	Specific goal setting (Goals and planning)	Promoter and participant agree short (weeks 1–3), intermediate (in one month) and longer-term (in three months) goals. Worked examples provided in employee booklet.
	Provide instruction (Goals and planning)	Promoter issues participants with booklet containing practical information, websites and a 10 week diary. Promoter booklet provides instructions on how to support the walkers.
	Provide general encouragement (Social Support)	Promoter, family, friends and work colleagues provide encouragement and affirmation.
	Self- monitoring of behaviour (Feedback and monitoring)	Participants asked to keep an optional record of walking behaviour in a diary. Promoter issues each employee with optional pedometer to monitor steps walked per day and can record steps in the diary.
2 (from week 3)	Techniques in contact 1 as appropriate	Participants encourage and support each other in changing their behaviour. Promoter offers assistance, encouragement, guidance and motivation to the employee. Participants encouraged to seek support from people outside the workplace such as family and friends.
	Plan social support (Social support)	
3 (from week 5)	Techniques in contact 1, 2 as appropriate	Promoter reviews intentions and short-, intermediate- and long-term goals to better suit the employee as necessary.
	Review of behavioural goals (Goals and planning)	
4 (from week 7)	Techniques in contact 1, 2, 3 as appropriate	Promoter identifies situations likely to result in participants readopting old behaviour or failure to maintain walking and helps plan to avoid or manage them recognising that it may take several attempts before walking to work becomes a habit.
	Relapse prevention (Goals and planning)	

The Walk to Work promoters’ were given packs which contained booklets for study participants that contained information about the benefits of walking to work and practical information on choosing routes, suitable footwear and clothing, maintaining personal safety, barrier identification and solutions, goal setting, use of self-monitoring and interactive websites. The booklet included diary pages to document the method of travel for the journey to and from work. Pedometers (Silva Sweden, step counter) were also provided for each participant to be used as an optional self-monitoring tool.

### Behaviour change techniques

The choice of the nine BCTs was influenced by the three categories recommended by NICE (goals and planning, feedback and monitoring, and social support) [16] and the systematic review conducted by Bird et al. [15]. The intervention focussed on nine BCTs taken from the Abraham and Michie [12] taxonomy: the BCTs, and the contact points at which they were used, are listed in Table 2.

During the first contact, where it is recommended that the promoter meet with the participant face to face, introducing themselves as well as issue the participant with the Walk to Work booklet and pedometer. The promoter and participant worked together to focus on the benefits of walking, identify barriers, propose solutions and develop a plan of how they might increase walking. This involved setting short, intermediate and long-term goals e.g. walking one day a week in the first week and then increasing it during the course of the intervention. They were also encouraged to complete diary sheets and record whether they had walked or not and, for those using pedometers, how many steps had been registered. The promoters were asked to make three further contacts with participants in person, by email or telephone, to suit the needs of the workplace and employees. Week three (contact 2) focussed on social support from other people such as colleagues, family or friends; week five (contact 3) stressed a review of goals to see whether they had been achieved or needed to be adjusted, and from week seven (contact 4) until the end of the intervention, the aim was to prevent relapse by supporting and encouraging participants to continue working towards their goals.

### Process evaluation interviews

We conducted interviews as part of the process evaluation to explore participants and promoters’ views and experiences of the intervention. A purposive sample was approached for interview based on workplace and gender. The sample included Walk to Work promoters, participants who reported an increase in walking to work in the post-intervention questionnaires, and participants who reported no increase in walking. The post intervention questionnaires were examined to determine those participants who self -reported a change in their travel. Semi-structured interviews were conducted by SP in a private room at the workplace of each participant between October 2012 and January 2013. Interviews lasted between 20 and 45 minutes. The topic guide allowed flexibility for participants to follow their own train of thought as well as including specific prompts. All interviews were audio recorded, fully transcribed and anonymized, and electronically stored in a secure folder. Here we focus on data from the Walk to Work promoters and those participants who attempted to use the BCTs to increase walking to work.

### Analysis

The Framework approach for data management was used to aid analysis [25–27]. Familiarisation with the dataset included reading and rereading the interview transcripts. Sections of text were extracted verbatim and entered into primary charts for each BCT by SP (an extract from a primary chart is given in Additional file 1: Table S1). Sections of text were initially coded in relation to benefits and barriers, with multiple codes allocated where appropriate. The primary charts were retained and revisited as required, but streamlined versions were produced as the process of summarising and synthesising the data progressed (Additional file 1: Table S2). In these subsequent charts, key terms and phrases were retained while repetition and extraneous text were removed by SP. During this process, differences or similarities were identified within emerging themes. SA scrutinised and commented on the coding and data interpretation. Differences were resolved through re-examination and discussion of the primary data.

## Results

### Participants

The flow of workplaces and participants through the study is shown in Figure 1. Following the distribution of information about the study, 187 participants consented to take part, of which 100 were in the intervention arm. Eight Walk to Work promoters were recruited from six of the workplaces. Of 16 participants approached for interview, 14 of them agreed, however two female participants from large workplaces E and F declined due to time commitments. The eight Walk to Work promoters were also interviewed. Details of the interviewees are shown in Table 3. Their ages ranged from 22 to 65 years, with a spread of household incomes, and 45% were female. All were in sedentary occupations, with most being city centre based. Each interviewee was given an alpha numeric ID to ensure participant confidentiality and anonymity: For example, interviewee F7 refers to workplace F and interviewee 7. The responses of participants are considered below in relation to each of the BCTs.

**Table 3:** **Characteristics of participants and Walk to Work promoters interviewed (all sedentary occupations)**

ID	Sex	Age	Participant and/or promoter	Workplace size ^1^	Type of business [28]	Location	Household income £k p.a.
A1	female	50	Participant/Promoter	small	Professional, scientific & technical	City centre	30-40
B1	female	26	Participant	small	Professional, scientific & technical	City centre	20-30
B2	female	33	Participant	small	Professional, scientific & technical	City centre	20-30
B3	male	22	Participant/Promoter	small	Professional, scientific & technical	City centre	>50
C1	male	29	Participant/Promoter	small	Professional, scientific & technical	Suburban	>50
C2	male	25	Participant	small	Professional, scientific & technical	Suburban	20-30
C3	male	65	Participant	small	Professional, scientific & technical	Suburban	Not given
C4	female	52	Participant	small	Professional, scientific & technical	Suburban	40-50
D1	male	60	Participant	medium	Professional, scientific & technical	City centre	>50
D2	male	38	Promoter	medium	Professional, scientific & technical	City centre	20-30
D3	male	37	Participant	medium	Professional, scientific & technical	City centre	>50
D4	female	52	Participant	medium	Professional, scientific & technical	City centre	Not given
E1	male	30	Promoter	large	Public administration	City centre	20-30
E2	male	52	Participant	large	Public administration	City centre	>50
E3	female	55	Participant	large	Public administration	City centre	30-40
F1	male	23	Participant	large	Financial & insurance activities	City centre	20-30
F2	female	58	Promoter	large	Financial & insurance activities	City centre	20-30
F3	female	46	Promoter	large	Financial & insurance activities	City centre	20-30
F4	male	46	Participant	large	Financial & insurance activities	City centre	30-40
F5	female	45	Participant/Promoter	large	Financial & insurance activities	City centre	>50
F6	female	31	Participant	large	Financial & insurance activities	City centre	30-40
F7	male	45	Participant	large	Financial & insurance activities	City centre	40-50

^1^ Size: Small <50; Medium 51–250; Large >250.

### Intention formation

When asked about intention formation, the participants focussed on their general targets for walking to work. For some this was the whole journey to and from work every day; others selected specific days of the week, either the journey to work or the journey home, or walking part of the journey and using the car or public transport for the remainder. Some justified these intentions for the health benefits such as *‘getting fit and losing weight’* (participant A1, female, 50). In two cases a doctor had recommended walking to alleviate medical conditions (high blood pressure and repetitive strain injury) and the study provided the opportunity to put this into practice: *‘My doctors said before that I need to, to walk more … I mean it’s the kind of thing that you sort of – you kind of know anyway really within yourself that you know you’re not doing enough of any kind of exercise. This [study invitation] is what gave me the stimulus if you like to actually get on and do something about it … and even the doctor didn’t even manage to persuade me of that’* (participant C4, female, 52). However, one promoter (F3, female, 46) observed that some people did not want to commit to a 10-week programme and, although people had good intentions, there was concern about dependency on use of cars *‘I think people [study participants] have the intentions of walking … but, because their character is, just they don’t know how to live without the car’*.

### Barrier identification

Interviewees discussed perceived barriers to walking, rather than the process of identifying barriers. The main barriers raised were the weather and motorised traffic: *‘The days when it’s raining isn’t quite so much fun but, apart from that, I mean crossing the roads is a bit of a nightmare sometimes especially if it’s raining and … you got to turn round and see if there’s a car and you’ve got to double check, wearing the glasses as well you know … you wouldn’t think there was a learning curve to crossing the road at my age but there is’* (participant C4, female, 52). Participant D1 (male, 52) raised the issue of the impracticality of wearing business suits in bad weather: *‘… turn up wet in the morning given the climate … you’re less inclined I think, if you walk, to wear any special clothing to walk in to work … normally people wouldn’t carry suits in their backpacks’*. Participant F6 (female, 31) was also reluctant to walk in the rain: *‘I did walk sometimes in the rain though, it wasn’t actually too bad once you’re in it, it’s just the thought of going out’*. Solutions proposed by participants tended to focus on being prepared for bad weather by having waterproofs and umbrellas: *‘I mean the weather this morning was horrendous but I’ve got some waterproof trousers, an overcoat and an umbrella and I came to work as dry as I would have been if it had been dry’* (participant D3, male, 37).

Having car parking at work was considered a barrier, and lack of car parking an enabler, to walking: *‘We haven’t really got limitations to car parking because we’ve got a free car park out the back so it is easier for us to drive because we know that we can park when we get here. If we didn’t have that then it would probably force us to walk a bit more – if you were in the centre or something like that I’d probably would have to walk or get public transport but because we’ve got free car park we can just come and go when we please in the car’* (participant/promoter C1, male, 29). Another barrier was a lack of facilities such as showers, changing facilities and lockers at work to support walkers: *‘If there were facilities or if you had that sort of stuff available to you I think I would be more inclined to walk or cycle, because I don’t – I don’t like feeling unclean, I like to be clean’* (participant C2, male, 25). This participant proposed a solution: *‘You’d have to like have a shirt ready in work so you’d have to make sure you prepare everything’*.

Participant C4 (female, 52) also identified a solution to the barrier she felt of arriving at work flustered and not being able to work straight away: *‘I’ve settled on walking home partly because that’s logistically how it’s mostly worked out anyway … I did walk in a couple of times and one time you know it looked like it was going to rain and I’m hurrying along hoping it’s not coming and I arrive in work and actually I didn’t feel as good when I arrived in work as I thought it would. I felt flustered and I felt as though I was behind myself and I didn’t feel as calm and you know. Whereas I drive, I park in the car park, I sit down at my desk and I’m straight into it’*.

The convenience and high fixed cost of cars were seen as barriers: *‘I’m paying so much for the petrol, the tax, the insurance I just think it’s there and it’s just convenient for me to use it so I think it’s just too easy to, to walk out the house, get in the car and just put my foot down and drive for 10 minutes up the road. Whereas if I walked I probably have to leave – maybe give it an hour just to make sure I was in time’* (participant C2, male, 25). The cost, time and unreliability of the public transport were important reasons why participants who lived further from the workplace chose to use their car, and ‘park and walk’ , rather than use the public transport system: *‘Public transport for me to try and get here is horrid. I tried it within the study, it was expensive, it will take me an hour and a half to get in here whereas by driving and walking I can do it in 35 minutes’* (participant F4, male, 46).

Caring responsibilities and time were highlighted as a barrier: ‘*They’ve got to pick up children from schools or clubs or some people have elderly relatives that they look after… some people will be rushing home to go off to yoga, pilates, dance so it depends if it’s going to take a lot longer*’ (promoter F2, female, 58). Other barriers included having to carry heavy items, needing the car for work appointments, walking in dark or unpleasant surroundings, pollution, shift work and the nature of the terrain: *‘I come down a very steep, two very steep hills … so walking in is not an issue, walking home is a bit more of an issue … it takes longer, it’s more tiring’* (participant E2, male, 52).

### Specific goal setting

The participants commended the Walk to Work booklets for providing *‘structure’* (participant E2, male, 52) and being *‘helpful’* (participant/promoter A1, female, 50) in encouraging them to think about short-, intermediate- and long-term goals. The goals reported by participants varied in their degree of specificity and detail. One enthusiastic participant (participant E3, female, 55) articulated clearly: *‘Short term to walk to [supermarket] which is specific place at a gentle pace in week one … second week was to do the same but at a faster pace and week three was to walk halfway once a week at a faster pace, a brisker pace … intermediate walk halfway to work twice a week … and then long term walk halfway to work every day which is about 25 minutes’.* Participant F6 (female, 31) was less precise: *I parked like close … then gradually sort of kept parking further back’*.

Pedometers assisted goal setting for seven participants: ‘*I just said to myself for the first two weeks I will just park my car where I normally park it and walk to work, do my stuff, go home and over the two weeks was just to see how many steps I was taking, and then I just said to myself over the next two/three weeks put it up another 1,000 steps and then kept increasing it, over the 10 weeks’* (participant F4, male, 46).

The role of the Walk to Work promoter was to work closely with participants to help them set their goals. Promoters varied in their degree of engagement with this goal setting and listening to the specific needs of the participant. Participant E3 (female, 55) reported that the promoter was too forceful: *‘The barrier I had with him was, was that I kept saying my goal isn’t to walk to work … I knew that at the end of a working day I often don’t finish until 6.30-7.00 the last thing I want to do then is walk for nearly an hour … I was never wanting to do more than walk halfway to work … that was my ultimate, and that for me would be success and he [promoter] kept giving me routes and to get here and I kept saying but I’m not doing that am I because that isn’t my goal and you need to support me with my goal rather than yours … if I set my goal on what he wanted, I wouldn’t have achieved it and then I would’ve failed so I wanted to be able to succeed in what I wanted to do’*. Promoter F3 (female, 46) had a more relaxed view and allowed participants to change goals depending on their weekly activities: *‘… don’t you worry about it, you don’t have to do the same amount every week. We all have busy lives so as long as you are walking every day if you can, that’s absolutely fine’*.

### Provide instruction

The promoter’s booklet outlined the role of the promoter and the BCTs for each of the four contacts. Overall the promoters found their booklet *‘was well set out’* (D2, male, 38) and helped them approach participants: *‘It was informative and useful and helped me set out what I needed to do, promote walking to work to the colleagues, and how to approach them and stuff, I thought it was quite good’* (participant/promoter B3, male, 22). This was reiterated by promoter E1 (male, 30): *‘It’s not too intimidating, it’s quite accessible, it’s got a, sort of a structure you can work through, when the walk to work promoter meets with the employees to discuss and set goals and review them’*. However, promoter D2 (male, 38) commented: *‘I think it was well set out and bit disappointed with myself getting out of sync and not following it properly because it guides you well but I lost sight of which week I was on and what I was supposed to be achieving’*. Participant/promoter C1 (male, 29) suggested that it would be useful to support and motivate the promoters themselves by having a *‘little newsletter each week like email a newsletter just to remind you about the entire thing and kind of give you more information to convince you to keep on doing it’*.

The participants’ booklets were praised by both participants and promoters for their structure and the information provided, particularly details of websites and route planning information: *‘I thought it was very helpful, I mean the websites at the back were very good, one of them in particular … the Walkit one, walking to work was brilliant’* (participant F4, male, 46). The exception (participant D4, female, 52) compared the booklet unfavourably with a previous walking intervention she had been involved in: *‘I didn’t find it very helpful really no, it was very, sort of repetitive and I better admit before I go any further that I’ve done the most brilliant scheme in the past. They had like online articles so that you could earn so many points educating yourself round health benefits’*.

### General encouragement

Most participants valued the encouragement they received from promoters: somebody being *‘enthusiastic’* and *‘showing an interest’* (participant D4, female, 52) or being encouraged and offering to walk together *‘She’s been very good at encouraging me to try different things … congratulating me … we’ll walk together when I was going to [local landmark] because she walks past there’ (*participant/ promoter F5, female, 45). Promoter F3 (female, 46) felt it was important to see participants trying their best and encouraging them even if they did not achieve their initial goals: *‘The people I am promoting they are doing their best even if they are not doing as much as they thought they were going to do. That is now in their mind and they will think twice … whether they really need the car or they can walk, and for me that is a fantastic achievement’*.

However, there were challenges for the Walk to Work promoters in fulfilling their role: *‘I was quite busy during the middle and that was one of the reasons why the third contact slipped … I did tend to slightly put it off and say well I’ll do it start of next week and then on one of those starts of next week I was off sick and by the time I got back into work I forgotten that I’d been planning to do it that week, and time just gets away from you sometimes’* (Promoter E1, male, 30).

Several participants suggested external health promoters could provide additional encouragement: *‘Somebody coming in from outside, say doing half an hour at lunchtime just doing a presentation about it or, you know, longer and getting people there and talking about that and saying ‘and we have our in-house person who you know if you want to talk to him, d’you wanna get encouragement from him/her’ that would be great but I think somebody coming in from outside actually would be a good idea’* (participant D1, male, 60).

Participant C4 (female, 52) felt it was important for management to show encouragement by allowing people time to get used to their new routine: ‘*They’re not going to get penalised if they are a little bit late in because they didn’t leave early enough while they’re getting the hang of walking’*.

### Self- monitoring

Self- monitoring was encouraged through the use of diaries and pedometers. The diary included space to record the method of travel, time taken and, if using a pedometer, the number of steps per day. Participants commented on the usefulness of a diary to provide structure, visual representation of progress and highlight inactivity: *‘It was just that structure of doing that every day and noticing that, no this week I haven’t actually walked as many times as I thought I might do, and so you know, tomorrow I’ll make sure I do’* (participant E2, male, 52). The diaries were also thought to be useful in assessing journey times: *‘… made me far more aware of what time I need to leave home to get here on time’* (participant D3, male, 37).

However, participant C4 (female, 52) preferred talking with colleagues: *‘… found myself you know doing the walking home without having written it down and you know having told several people – I mean telling people that that’s what you’re doing actually makes you hold to it even more than if you, if I’d written it down’*. One participant found diary completion repetitive and time-consuming, while another found his initial enthusiasm tended to diminish with time ‘*I used it a few times to start with but then sort of fade out*’ (participant/promoter B3, male, 22). However, the diary could be helpful for promoters in monitoring how participants were progressing: *‘Every time I meet with them I just have a quick look [at the diary]’* (F3, female, 46).

Promoter C1 (male, 29) praised computer applications to aid self-monitoring: *‘I thought the apps and all the stuff that you do on the computer to be able to track and log everything and find the easier routes to walk and things like that was really, really good … I think they should be publicised a bit more because I definitely think that would help people and convince more people to walk’.*

Although pedometers were offered free of charge, seven of the participants chose not to use them, seven participants used the pedometers within goal setting aiming to increase their steps per day and the remaining four commented on problems using them: *‘I’ve not been very good at using a pedometer because I’ve either forgotten to put it on in the morning and I find them quite uncomfortable’* (participant/promoter F5, female, 45). One promoter (E1, male, 30) expressed his concern at asking participants to wear a pedometer for the duration of the intervention: *‘Ten weeks is a long time to be wearing such a device’*.

However, there was evidence that the step count could be motivating: ‘*We were probably hitting on a day when we weren’t walking an average of about 3,000 … it’s just a bit of a shock … I used the pedometer which I found really interesting, just to see how little we actually did walk when using the cars and how much more you actually do just walking to and from work’* (participant/promoter C1, male, 29).

### Social support

There were mixed feelings about the level of support offered by the Walk to Work promoters. Some participants felt the promoters were well placed to help them, particularly if the promoter had changed their own behaviour, had local knowledge, and was able to discuss barriers and support participants who needed reminding. Participant E3 (female, 55) expected her promoter to check progress more rigorously: *‘I expected we were going to go through this and he was going to go “Oh look you took a bit of a dip there [looking at diary]” … he just wrote to me and said “How you doing?”, and I wrote back and said “It’s going fine” and that was it really. Yeah I’m not sure what else he could have done I suppose but it did – it felt very light touch … I think I was expecting a bit more … kind of looking at this together and maybe another meeting’*. Another participant reported little or no contact from the Walk to Work promoter after the initial session: *‘I must admit in the 10 weeks they didn’t … they initially set you up yeah but no one to sort of halfway through said have you increased’* (F4, male, 46).

In some workplaces, participants offered mutual support, particularly if they were sitting close to each other: *‘We would always be having a conversation, there would always been a time when he would say “Oh I walked to work today, what did you do?”* (participant C2, male, 25). Such support included shared experiences of bad weather: ‘*It was quite nice that there was other people that get drenched when you get drenched*’ (B1, female, 26). In contrast, participant E2 (male, 52) had the support of his promoter but was not aware of anyone else taking part in the study: ‘*It might’ve made a difference if I actually knew somebody else who was doing it’*.

The promoters reported it was not always easy to support walkers particularly if there was work pressure or a more junior member of staff was supporting a manager: *‘One of the walkers is a manager … he’s way above my pay grade … I felt like I was demanding his precious time to meet with him to do the study, although of course he volunteered for it but … it was slightly strange relationship for me to be the sort of the mentor to someone whose on a sort of higher grade, managerial role’* (E1, male, 30).

### Review of behavioural goals

Participants appeared willing to reconsider their initial goals, particularly if they had been easily achievable, and to increase the amount and frequency of walking: *‘I reviewed myself and found that I can actually walk to work a lot more than I thought I could so I just upped the amount of times that I actually walked’* (participant/promoter C1, male, 29). A change in seasons had the potential to affect behaviour: *‘I did a little bit [review goals] when it first started getting really dark like in the mornings and evenings, and then when the clocks went back it kind of evened it out a bit better so I could sort of go back to finishing at six and feel OK about walking back’* (participant F6, female, 31). Promoter D2 (male, 38) highlighted the experience of one participant who reviewed his goals as he was concerned about the safety of the area he walked through. However, as there was no alternative, he continued with that route: *‘He thought, because he’s near the [local landmark], he’s got to walk through [local area], probably not safe, but there’s no other route you know, but he risked it anyway’*.

### Relapse prevention

One promoter suggested the term ‘relapse prevention’ was probably not appropriate for an intervention that increased walking to work: *‘Relapse prevention sounds like a stop smoking campaign. It’s a bit drastic for someone who has stopped walking’* (participant/promoter C1, male, 29). However, he acknowledged the need to give people tools to help them maintain their new behaviour: *‘It’s almost like having an internet site that you could go to for advice and stuff … like a little push just to say look you can still keep doing this if you do this, this and this’*.

Participant C2 (male, 25) suggested it was difficult to continue walking after a holiday but he was able to build up gradually after his break: *‘I went on holiday for two weeks so when I came back I was a little less inclined to be walking, but eventually I got myself back on track’*. He was also anxious that he was having a knee operation and this would cause him to resort back to using the car: *‘I’ve got my operation next week I’m off for six weeks then so I’m going to be on my bum a lot (laugh) so I can see me getting lazy over the winter period’*.

### Suggestions for change

There were some suggestions for introducing further BCTs not used in the intervention which participants felt would help them change their behaviour. Two promoters and five participants felt that the intervention would benefit from incentives: ‘*Offering a cash incentive for people to walk rather than drive*’ (participant F7, male, 45). Other incentives discussed included training shoes, vouchers or a free breakfast to encourage them to walk to work. *‘I think more of an incentive that, you know, if we got people to walk to work they got a free breakfast when they came to work or they you know if they started out earlier to walk to work well then there’d be some sort of just a little something, you know, people might think oh actually if I leave half an hour earlier and I walk into work, alright it’s two miles there might be a, you know, something or a voucher for a coffee place or something, breakfast bap or something’* (participant /promoter F5, female, 31).

Participants also talked about introducing competition between different workplaces taking part in the scheme: *‘An element of competition may help … if the progress was recorded and shared between all the people on the scheme, it could possibly have a positive effect … for people being able to walk the most or the greatest number of times’* (participant C3, male, 65). Participant B2 (female, 33) talked about competition within the workplace: ‘*I sit predominantly around a load of lads they are very competitive so maybe some kind of competition maybe something with the pedometers where you can measure how far everybody walks each day’*.

## Discussion

### Behaviour change techniques

The Walk to Work intervention focussed on nine BCTs to encourage a sustained increase in walking during the journey to or from work by participating employees. The BCTs were chosen to take the participant through the 10-week intervention: intention formation, barrier identification, goal setting, general encouragement, instruction, self- monitoring, social support, review of goals and relapse prevention. For each individual BCT, there appeared to be people who found it useful and others who did not. Other studies confirm this lack of clarity over which BCTs are essential for a behaviour change intervention [29]. This is in line with evidence from a qualitative evidence synthesis of workplace smoking interventions which argues that workplace interventions should employ a range of different elements if they are to prove effective because different employees have different requirements [30].

The concept of setting behavioural goals is identified as being important by both Bird et al. [15] and Malik et al. [29]. The ‘intention formation’ BCT has been updated from the 2008 version [12] to the 40 item 2011 taxonomy [13] to ‘goal setting (behaviour)’ and is defined in the paper as a behavioural resolution that does not involve planning when only ‘goal setting’ is mentioned in a study. Williams et al. [31] argues that setting a detailed plan of when, where and how to perform the behaviour was more effective at bringing about positive change in self-efficacy and physical activity behaviour than setting a general goal or make a general intention.

Additional motivation may have been helpful for some participants through providing ‘contingent rewards’ , also listed in the taxonomy of BCTs [12]. This could be facilitated by providing additional guidance for employers about giving small incentives, such as free breakfast or help to purchase walking shoes, for employees who change from commuting by car to walking. Other suggestions included a degree of competition, which may be motivating for some participants but off-putting for others. The BCT, ‘provide feedback on performance’ which evaluates performance in relation to others (or a set standard) could be explored [12].

This study used pedometers as an optional self-monitoring tool and participants had mixed views about using them. Although some found them important for goal setting, over half did not use them for practical reasons. Pedometers have been proposed as the single most effective method of physical activity promotion [32] offering participants an opportunity to goal set and self-monitor, improve self-efficacy and motivation, and increase social support by providing a visual prompt for use with promoters, family and friends [18, 33, 34]. McKay et al. [35] found group support and help from health professionals motivated participants to increase their step counts and this finding was shared by the Lauzon et al. [34] study of participant experiences of a workplace pedometer-based physical activity program. However, as with other BCTs, the current study suggests that pedometers were useful for some participants and not for others. It would seem that to develop an intervention that specifically requires their use may restrict the involvement of some potential participants.

The BCT ‘relapse prevention’ , which was used in Walk to Work booklets, was challenged as being inappropriate terminology for an intervention that promotes walking and that it did not need to be described that way in the intervention materials. This view is shared by Michie et al. [13] who argue that different BCTs should be used for initiating positive behaviours, such as physical activity and healthy eating, from those used to stop negative behaviour such as smoking and alcohol consumption. A term that focuses more positively on maintaining new behaviour may be preferred. The BCT ‘Use follow-up prompts’ [12] may be preferable as many people try to change behaviour several times before it becomes a habit through continued repetition of the new behaviour [36].

### The role of walk to work promoters

The Walk to Work promoters were either volunteers or were asked by their employer to take on the role, and showed varying interest in active travel. Although they all received training to understand and use the nine BCTs, this was also approached with varying levels of enthusiasm: most appearing keen to help and generally encouraging, one perhaps being over enthusiastic and another struggling to fulfil the role because of other work commitments. Where one promoter was of a ‘lower grade’ than the colleague they were supporting to walk to work, there was evidence of uncertainty about how to deal with this ‘role reversal’.

Similarly, participants also varied in their reflections on the support provided by promoters, from a feeling that it was too ‘light touch’ to concern that they were being pressurised into meeting unrealistic expectations. This would suggest that training of promoters should include awareness of how to assess and respond to the varying needs of participants. The availability of a range of approaches to providing support, including face-to face contact with a promoter, guidance booklets, self- monitoring equipment and interactive websites may help to ensure that the different preferences of participants can be catered for.

A review undertaken on behalf of NICE [16] identified a number of characteristics and competencies for those delivering BCT interventions which included being supportive, motivating and empathetic, as well as having knowledge and communication skills associated with assessing individuals, signposting support, developing motivation and action, providing feedback, action planning, goal setting and problem solving, encouraging self- management, group counselling and maintaining change and relapse prevention. It is unrealistic to expect volunteers in the workplace to have all of these characteristics and competencies, together with the time and space to implement them. For the Walk to Work intervention, the promoters were provided with a half-day training session supported by booklets and signposts to websites and other useful resources. The appropriate use of these resources, combined with a supportive and empathetic approach, may be an appropriate compromise for a workplace public health intervention of this kind. Nevertheless, workplace volunteers who undertake this role may require some additional training to develop skills and competencies, or external support from those with greater knowledge and experience of health promotion interventions [16, 37].

### The socio-ecological model

Advocates of the socio-ecological model argue for the importance of examining influences at the policy, community, organisational, interpersonal and intrapersonal levels [9]. BCTs are primarily focussed at the intrapersonal and interpersonal levels, but this study highlights the importance of support at the other levels of the model, particularly the workplace (organisational level) through, for example, employers providing free breakfast for walkers or improved facilities to wash and change. The availability of car parking was identified as an important barrier; a finding echoed by others [11, 38, 39] who suggest that limiting workplace parking may encourage walking to work. For example, a workplace Travel Plan at the University of Bristol, which included controlling the supply and cost of car parking spaces for staff, resulted in a self-reported increase in walking to work from 19% to 30% and a reduction in commuting by car from 50% to 33% [40]. In the current study, environmental barriers such as unpleasant or potentially unsafe routes were mentioned but were not as prominent as in other studies [11, 38, 39]. This may support the view of Guell et al. [38] who point out that some participants will walk despite adverse environmental conditions, having overcome the issue through experience or weighing up the perceived benefits and costs. The problems of a hilly terrain could be addressed by choosing to walk only the downhill route to or from work, and could be considered as a ‘solution’ [41].

### Wider contextual issues

It is also important to acknowledge the contextual issues which may undermine behavioural change interventions and two important factors are likely to have influenced the actions and attitudes of participants and the recruitment of participants to the intervention. Firstly, the study took place in the aftermath of a global banking crisis [42]. This resulted in economic insecurity, with businesses restructuring and an increase in unemployment. Under these circumstances, health promotion interventions may be seen as less important to employers and employees than the survival of businesses and the retention of jobs. Secondly the intervention was implemented during the wettest summer in the UK for 100 years [43]. Weather conditions have been identified as an important barrier to walking and this is likely to have been exacerbated by the weather during the summer of 2012 [24, 44]. However, even within this context, there was evidence of willingness to consider the BCTs and change travel behaviour.

### Strengths and limitations

The strengths of the study include using a qualitative approach to discover participants’ views, the spread of male and female participants of different ages, income groups and from small, medium and large workplaces and the inclusion of promoters as well as the participants. The topic guide sought to gain views on each of the BCT’s used in the intervention but also allowed people to tell their story in their own way and to emphasise what was important to them.

All of the authors were involved in developing the intervention; SP, SA and AD trained the Walk to Work promoters, and; SP undertook the interviews. Therefore, in analysing the data and preparing this manuscript, the authors were particularly conscious of the need to avoid unwarranted positive appraisal. Furthermore, it was emphasised to interviewees that this was a feasibility study attempting to assess the acceptability of the intervention which facilitated a degree of candidness about the perceived weaknesses, as well as any strengths, of the intervention.

Other limitations of the study include a relatively small sample size of predominantly city-centre based participants, all in sedentary occupations. The interviewees were, inevitably, all consenting participants in a research study and their views may not be representative of the wider workforce. Additionally, the interviews took place in the workplace and while this was convenient, it meant that some interviewees were aware of the need to return to work and unable to explore issues in depth.

As the main study was a feasibility study, the topic guide covered a broad range of questions about the intervention and its evaluation. Despite being asked about each BCT used in the intervention, participants often did not talk specifically about the technique or related strategies. Some BCTs may have appeared too abstract for participants to comment on, hence their tendency to talk about the more concrete aspects of their experiences of the intervention. A future qualitative study might benefit from focussing more specifically, and in more depth, on the use of BCTs.

## Conclusion

Walk to work interventions employing BCTs should include sufficient techniques to enable participants to choose a ‘package’ to suit their individual needs. Additional support at organisational level should also be encouraged, and consideration given to wider contextual factors that impinge on the delivery of, and response to the intervention.

## Electronic supplementary material

Additional file 1: Table S1: Primary chart for self-monitoring BCT. **Table S2.** Coded and reduced table for self-monitoring BCT. (DOCX 17 KB)

## Abbreviations

BCT: Behaviour change technique
NICE: National Institute for Health and Care Excellence
SD: Standard deviation.
