# Supplementary material for: Views and experiences of behaviour change techniques to encourage walking to work: a qualitative study
Source: BMC Public Health. 2014 Aug 23;14:868. doi: 10.1186/1471-2458-14-868 (PMC4158136; doi:10.1186/1471-2458-14-868)
Supplement: Supplementary file 1 — Additional file 1: Table S1: Primary chart for self-monitoring BCT. Table S2. Coded and reduced table for self-monitoring BCT. (DOCX 17 KB) [file 12889_2014_7011_MOESM1_ESM.docx]

**Table S1: Primary chart for self-monitoring BCT**

| **Study ID** | **Details** | **Self monitoring** |
| --- | --- | --- |
| A1 | Promoter  Sex: F  Age: 50 | A1: I liked the fact there’s a diary, and it really takes you through what to do and like you write everything down, which I think really helps and setting goals I think is … And writing down what, overcoming the barriers, that’s a good idea as well. I think all that’s good.  INT: Yeah. OK thank you, did you record your progress in the back of the diary?  A1: I don’t believe I did, no. Sorry about that.  INT: Yeah. Can you explain why?  A1: Oh, um, time? I suppose.  INT: Yeah  A1: Lack of time, I think.  INT: Yeah, and did you use the pedometer?  A1: I haven’t, I haven’t used the pedometer no.  INT: No. We suggest it as an aid.  A1: OK yeah, OK.  INT: So it’s not, it’s, some people find that monitoring is something that works for them, and other people don’t.  A1: I decided it was another thing yes, yeah. So, yeah, so I didn’t use it, no. |
| B1 | Sex: F  Age: 26 | INT: Did you record your progress in the, in the diary at the back?  B1: Yes, I did yes.  INT: Yeah. And did you find it helpful?  B1: Um yeah I did yeah, yeah it was quite interesting to see like how you get to work and how long it takes you, whereas sometimes you’re just on auto pilot just like meandering along so was quite nice to – “Oh yeah that only took me ten minutes” or “Oh did it in fifteen, what was I doing?” sort of thing [I: yeah] so yeah that was quite good.  INT: We provided you with pedometers. Did you use the pedometers at all?  B1: No I didn’t, no I forgot about them – sorry. |
| B2 | Sex: F  Age: 33 | INT: Did you record your progress at the back in this diary?  B2: Yeah. That was quite interesting and it was you know if I didn’t use my car at all for anything it was quite nice to have all the walks like so (laugh) ”God look how good I am”.  INT: Good. And did you use the pedometer at all?  B2: Yeah.  INT: Yeah. And um how did you get on with that?  B2: Can’t remember how many steps it was now to and from. Um but yeah again it was, it was quite interesting to see you know how far it was and then compare it with other trips that I was making and things.  INT: So good and um did you find it helpful um knowing the steps?  B2: I [[hesitant]] yes and no really, I mean it was I think the first sort of couple of days is quite interesting to go ‘oh so it’s that many’, but because I do it all the time I think I kind of it’s like oh it’s going to be that again kind of thing. |

INT = Interviewer

**Table S2: Coded and reduced table for self-monitoring BCT**

| **Study ID** | **Details** | **Self monitoring** | **Code** |
| --- | --- | --- | --- |
| A1 | Promoter  Sex: F  Age: 50 | A1: I liked the fact there’s a diary, and it really takes you through what to do and like you write everything down, which I think really helps and setting goals I think is … And writing down what, overcoming the barriers, that’s a good idea as well. I think all that’s good.  INT: Yeah. OK thank you, did you record your progress in the back of the diary?  A1: I don’t believe I did, no. Sorry about that ...  INT: Yeah. Can you explain why?  A1: Oh, um, time? I suppose.  A1: Lack of time, I think.  INT: Yeah, and did you use the pedometer?  A1: I decided it was another thing yes, yeah. So, yeah, so I didn’t use it, no. | Benefit: Diary/ helpful write down/goal setting  Barrier: Diary/time constraints  Barrier: Pedometer /time constraints/  ‘Another thing’ |
| B1 | Sex: F  Age: 26 | INT: Did you record your progress in the, in the diary at the back?  B1: Yes, I did yes.  INT: Yeah. And did you find it helpful?  B1: It was quite interesting to see like how you get to work and how long it takes you, whereas sometimes you’re just on auto pilot just like meandering along so was quite nice to – “Oh yeah that only took me ten minutes” or “Oh did it in fifteen, what was I doing?” sort of thing so yeah that was quite good.  INT: We provided you with pedometers. Did you use the pedometers at all?  B1: No I didn’t, no I forgot about them – sorry. | Benefit: Diary/ timings to work  Barrier: Pedometer /forgot to use |
| B2 | Sex: F  Age: 33 | INT: Did you record your progress at the back in this diary?  B2: Yeah. That was quite interesting and it was you know if I didn’t use my car at all for anything it was quite nice to have all the walks like so (laugh) ”God look how good I am”.  INT: Good. And did you use the pedometer at all?  B2: It was quite interesting to see you know how far it was and then compare it with other trips that I was making and things.  INT: So good and did you find it helpful um knowing the steps?  B2: I [[hesitant]] yes and no really, I mean it was I think the first sort of couple of days is quite interesting to go ‘oh so it’s that many’, but because I do it all the time I think I kind of it’s like oh it’s going to be that again kind of thing. | Benefit: Diary/ record when not using car  Benefit: Pedometer / compare steps with other trips  Barrier: Pedometer /repetitive after couple of days |

INT = Interviewer
